# Histopathological Features of the Colon in Chronic Strongyloidiasis: A Case Series

**DOI:** 10.7759/cureus.90936

**Published:** 2025-08-25

**Authors:** Kyoko Arakaki, Kayoko Higuchi

**Affiliations:** 1 Diagnostic Pathology, Okinawa Kyodo Hospital, Naha, JPN

**Keywords:** chronic strongyloidiasis, colitis, colon, colonoscopy, histopathology, strongyloides stercoralis

## Abstract

Strongyloidiasis, which is caused by *Strongyloides stercoralis *(a* *nematode), is a prevalent human intestinal parasitic disease, particularly in tropical and subtropical regions. Okinawa Prefecture exhibits the highest prevalence of strongyloidiasis in Japan. This nematode has the unique ability to replicate for years within a human host with minimal or no symptoms, a phenomenon known as autoinfection. Patients with *Strongyloides stercoralis* autoinfection are often asymptomatic or experience uncomplicated infections, and, hence, are considered to have chronic strongyloidiasis. In this study, we retrospectively investigated the characteristic colonoscopic and histopathological findings of the colon in patients with chronic strongyloidiasis.

From 2004 to 2023, 22 almost asymptomatic patients underwent colonoscopy as part of routine health check-ups at one of three centers in Okinawa, Japan.

The subjects’ ages ranged from 53 to 87 years. The male-to-female ratio was 17:5. Colonoscopy showed yellowish-white nodules on the cecum and the proximal ascending colon. Biopsy specimens revealed filariform *Strongyloides* larvae with small, dense infiltrations of eosinophils in the lamina propria.

The findings presented in this study are useful for diagnosing chronic strongyloidiasis and suggest that the re-invasion sites of filariform larvae may be located in the cecum and proximal ascending colon.

## Introduction

*Strongyloides* *stercoralis*, an intestinal nematode, was discovered in the stools of soldiers returning to France from Southeast Asia in 1876 [1,2]. It is widely distributed throughout tropical and subtropical regions of the world. In Japan, the inhabitants of the southwestern islands, including the residents of Okinawa Prefecture, exhibit the highest rates of *Strongyloides stercoralis* infection [3-5]. Although the infection rate has decreased in recent years due to improved sanitary conditions, sporadic outbreaks of hyperinfections and disseminated infections still occur, and some deaths have been reported [6-9].

It is generally thought that filariform *Strongyloides* larvae in soil enter the body percutaneously, migrating through the circulation to the lungs and then to the upper small intestine, where they mature into adult worms. In infected individuals, larvae, adult females, and eggs are found in the crypts of the upper small intestine [5,10]. Worms and eggs are excreted in stools or die, which often ends the infection [2]. However, some filariform larvae within the intestinal tract may penetrate the bowel mucosa or the perianal skin and retrace their predecessors' migratory paths [2,10-12]. In this way, *Strongyloides stercoralis* can survive for many generations in the body of an affected person while causing no or minimal symptoms [2,11,12]. This mode of infection is called autoinfection [2,11,12]. Patients experiencing *Strongyloides stercoralis* autoinfection are often almost asymptomatic and are considered to have chronic strongyloidiasis [11-13]. Diagnosing chronic strongyloidiasis remains challenging [11,12]. Stool examinations based on agar-plate cultures and upper gastrointestinal examinations, including biopsies, may be useful for diagnosing strongyloidiasis [4,14-16]. But these methods are not routinely employed for asymptomatic patients. However, diagnosing chronic strongyloidiasis is crucial for preventing fatal disseminated strongyloidiasis.

We previously described 10 cases of chronic strongyloidiasis in 2011 [5]. In this prior study, we demonstrated the characteristic colonoscopic findings of patients with chronic strongyloidiasis, i.e., yellowish-white nodules in the right colon, as well as the typical histopathological findings of biopsy specimens from such lesions, i.e., filariform larvae within eosinophilic infiltrates.

In the present study, 12 additional cases were included to enable a more detailed histopathological analysis. We re-examined the endoscopic and histological findings of these cases and discussed the re-entry sites used by larvae for autoinfection.

## Materials and methods

The cases of 22 patients who had been diagnosed with strongyloidiasis based on colonoscopic screening during health check-ups conducted between 2004 and 2023 were retrospectively reviewed. The patients underwent colonoscopy at one of three hospitals in Okinawa, Japan (cases 1-16: Tomishiro Central Hospital, cases 17-21: Naha City Hospital, case 22: Okinawa Kyodo Hospital). Endoscopy was performed with forward-viewing endoscopes. Biopsy specimens were obtained from colonic mucosa sites with abnormal findings in all patients using standard forceps. Hematoxylin-eosin staining was attempted in all cases for histopathological evaluation. In the histopathological evaluation, a single researcher (the first author) evaluated the biopsy specimens alone. We focused on the following points: the presence/absence of parasites, the shape and size of any parasites (adult or larva), the presence/absence of inflammation, the location and extent of inflammation, and the types of inflammatory cells present. Adult worms in the duodenal mucosa of another patient were also stained with hematoxylin-eosin staining as a reference. Fecal culture testing was performed using the agar plate culture method as follows. A few grams of feces were placed on agar medium and incubated at 37°C for more than 24 hours. The presence of larval crawling tracks or unique bacterial colonies developing along the crawling tracks was considered to indicate a positive result. All of the study participants provided informed consent, and the study design was approved by the Ethics Review Board of Naha City Hospital (approved number 2023a31).

## Results

The clinical information of the 22 patients and the affected intestinal regions are summarized in Table 1.

**Table 1:** Clinicopathological findings of 22 cases EP: blood eosinophilic count, n.d.: not determined, HTLV-1: human T-lymphotropic virus type 1, C: cecum, A: ascending colon, T: transverse colon, S: sigmoid colon

Case number	Age	Sex	Symptoms	EP/μl	HTLV-1	Clinical condition	Affected region	Stool exam.
1	70	M	None	572	Negative	Gastric cancer, preoperative state	C	Positive
2	75	M	Epigastric pain	n.d.	Negative	None	C, A	n.d.
3	68	M	None	705	Positive	Right hemicolectomy	T, S	Positive
4	71	M	None	n.d.	n.d.	Chemotherapy for lung cancer	C	n.d.
5	87	F	Epigastric pain	6020	Negative	Asthma	A	Positive
6	73	M	Mild weight loss	699	Positive	None	C, A	n.d.
7	71	M	Abdominal fullness	130	Positive	Laryngeal cancer, postoperative state	C, T	Negative
8	69	M	None	302	Positive	None	C	Positive
9	74	M	None	n.d.	n.d.	None	C	Positive
10	78	M	None	1375	Negative	None	C	Positive
11	74	M	None	2589	n.d.	Undergoing follow-up for colon cancer	C, A	Positive
12	56	F	Soft stools	n.d.	Positive	None	C, A	n.d.
13	60	M	Abdominal fullness	n.d.	Positive	None	C	n.d.
14	62	M	None	n.d.	Negative	None	A	Negative
15	53	F	None	n.d.	n.d.	Nephrotic syndrome	C	n.d.
16	76	M	None	n.d.	Negative	None	C	Negative
17	62	M	None	n.d.	Positive	None	C, A	n.d.
18	81	M	None	1940	Negative	Intraabdominal tumor	C, A, S	Positive
19	82	M	None	530	Negative	None	C	Positive
20	84	F	None	4482	Negative	None	C	Positive
21	83	F	None	1344	Positive	None	C, A	Negative
22	74	M	None	1100	Negative	None	C, A	Negative

We previously published 10 cases (cases 1-10) [5]. In this report, we present an additional set of cases (cases 11-22).

The patients’ ages ranged from 53 to 87 years (mean: 72 years). The male-to-female ratio was 17:5. Sixteen patients (73%) were asymptomatic, and six (27%) had mild symptoms, including two with epigastric pain, one with weight loss, two with abdominal fullness, and one with soft stools. Eosinophilia was present in 11 of 13 patients who were tested (84%). Eight of 18 (44%) patients who were tested were HTLV-1 (human T-lymphotropic virus type 1) carriers. Eight patients (36%) had underlying diseases: three had a suspected malignancy, one was undergoing postoperative chemotherapy for lung cancer, two were undergoing follow-up evaluations for colon cancer, and two were receiving steroid treatment. The steroids were being administered for nephrosis and asthma. Stool examinations were performed in 15 cases and resulted in positivity for *Strongyloides* in 10 (67%) cases.

The lesions found during colonoscopy were generally confined to the right colon, with the cecum being the most common site, followed by the proximal ascending colon. The cecum was affected in 19 patients, whereas the ascending colon was affected in 10 patients, the transverse colon was affected in two patients, and the sigmoid colon was affected in two patients. The two patients with sigmoid colon infections were cases three and 18. Case 3 underwent a right hemicolectomy and ileum-transverse colon anastomosis, and the lesions were located in the transverse and sigmoid colon. In case 18, lesions were scattered in multiple areas (the cecum, ascending colon, and sigmoid colon). On the other hand, the ileum was almost intact in all cases (Table 1).

**Figure 1:**
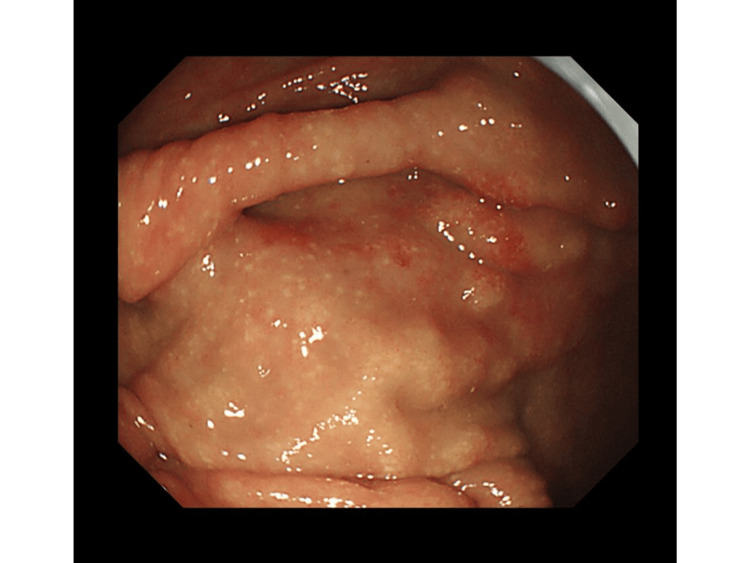
Colonoscopic findings Yellowish-white nodules were seen in the cecum (a) (case 11).

Colonoscopy revealed yellowish-white nodules in 17 of 22 patients (77%) (Figure 1).

**Figure 2:**
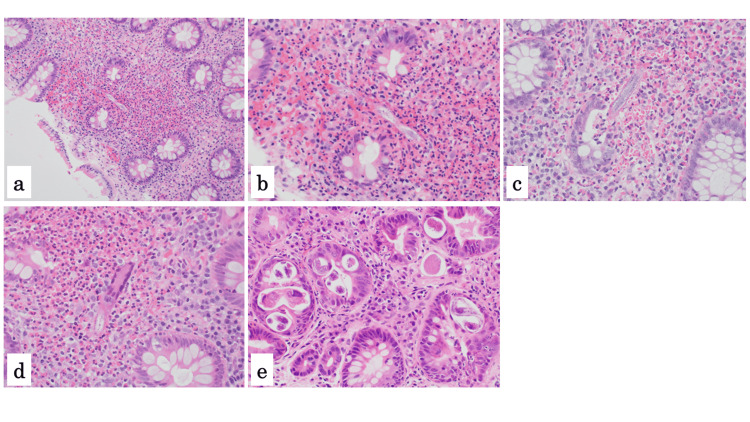
Histology of colon biopsies a, b: Case 11; c: Case 19; d: Case 11; e: duodenal biopsy (HE-stained specimens). Eosinophilic-dominated inflammation, involving lymphocytes and neutrophils, was seen in the mucosa (a, b). Larvae were identified within the eosinophilic regions (a-d). Microgranules were present inside the worms (b). A larva was observed escaping from a crypt and into the lamina propria (c). Larvae were phagocytosed by multinucleated histocytes (d.) Adult worms were seen in the duodenum (e).

A total of 14 (63%) had yellowish-white nodules alone, three (14%) had both yellowish-white nodules and erythema, and five (23%) had erythema alone. Strongyloidiasis was diagnosed pathologically in all 22 patients, based on the presence of filariform *Strongyloides* larvae and small, dense eosinophilic concentrations in the lamina propria with crypt distortion (Figures 2a, 2b).

All biopsy specimens obtained from the colonic lesions exhibited an eosinophil-dominated inflammatory response, involving lymphocytes and neutrophils, in the lamina propria. The larvae were about 10 µm in width and 70 µm in length, and their bodies contained several microgranules (Figure 2b). Larval migration from the gland crypts into the lamina propria (Figure 2c) and the phagocytosis of larvae by multinucleated giant histiocytes (Figure 2d) were observed. As a reference, Figure 2e shows adult worms in the duodenum of another patient (Figure 2e).

The patient responded well to treatment and has a favorable prognosis. There were no significant findings suggestive of malignancy, inflammatory bowel disease, or vasculitis in the biopsy specimens.

## Discussion

Strongyloidiasis, which can affect both immunocompromised and immunocompetent individuals, occurs in three forms: acute infections, chronic infections, and hyperinfection/disseminated infections [11,12]. Acute infections and hyperinfections/disseminated infections vary from mild to severe in symptom presentation. Chronic infections are generally asymptomatic but may occasionally present with mild gastrointestinal symptoms [11,12]. In this study, we aimed to present the findings of chronic strongyloidiasis involving colonic lesions.

The worms we found in the colonic mucosa in this study were minute and ultrafine (70 µm in length and 10 µm in width). Their internal structures were immature, and they only contained a few microgranules. They invaded the lamina propria, causing small, dense eosinophilic infiltrates with crypt distortion. They were suspected to be filariform *Strongyloides* larvae with the ability to invade the human body [2,5,10]. We observed that most infiltrating larvae were damaged by eosinophils and eventually phagocytosed by multinucleated histiocytes. We suspect that only the surviving larvae were capable of reinfecting the host. The pathological features observed in the colon are very different from those seen in the duodenum. Adult worms, which solely inhabit the duodenal gland crypts, were larger than the filariform larvae in the colonic mucosa, and the inflammation of the duodenal mucosa was mild.

Most reported cases of strongyloidiasis with colonic lesions were cases of hyperinfection or disseminated strongyloidiasis [6-9]. However, we previously reported that cases of chronic strongyloidiasis may exhibit colonic involvement [5]. The present study provides further evidence to support this.

Although all of the cases reported here involved larvae being found in the colonic mucosa, even if larvae are absent, the presence of eosinophilic concentrations in the right colonic mucosa on pathology specimens should raise suspicion of strongyloidiasis in endemic regions. In our study, the most common sites of larval infiltration were the cecum and proximal ascending colon, while the distal end of the ileum was confirmed to be free of lesions. Samman M AI et al. also described a normal ileum in a case of *Strongyloides*-related pancolitis [13]. In case 3, in which the patient had previously undergone surgery for ascending colon cancer and the ileum and transverse colon had been anastomosed, the lesions were located distal to the anastomosis. In case 18, multiple lesions were scattered throughout the cecum, ascending colon, and sigmoid colon. In the cecum, the lesions were mild, while in the sigmoid colon, erosions and ulceration were seen. In this case, there is a possibility that the infection has progressed somewhat. Although we did not examine the entire small intestine, these findings led us to speculate that filariform larvae may initiate their re-invasion into the patient's body as they pass through the small intestine and reach the colon.

Clinically, this study showed that chronic strongyloidiasis is common in elderly males, with 73% being asymptomatic and 27% being mildly symptomatic. Cancer patients and steroid users accounted for 36% of cases, while Human T-cell lymphotropic virus type 1 (HTLV-1) carriers accounted for 44%. HTLV-1 carriers are known to exhibit a high rate of strongyloidiasis [17-19]. These findings may suggest the involvement of a mild immunosuppressive state. Eosinophilia was observed in 11 (84%) of 13 tested cases, and in one case (case number 20), unexplained eosinophilia led to the diagnosis of asymptomatic strongyloidiasis [11,12].

Pathologically, there are two differential diagnoses to consider: eosinophilic enterocolitis and anisakiasis. Eosinophilic enterocolitis is the leading differential diagnosis for eosinophilic infiltrative lesions in the colonic mucosa. In cases of strongyloidiasis, eosinophils present as small, dense, regional infiltrates. On the other hand, in eosinophilic enterocolitis, the infiltration is generally diffuse, rather than being densely clustered in small areas [20-22]. Distinguishing between the two conditions is important because steroid administration under a diagnosis of eosinophilic enterocolitis in patients with strongyloidiasis may lead to a fatal hyperinfection [6]. The incidence of hyperinfection and disseminated strongyloidiasis has been reported to range from 0.5 to 4.7% of infected individuals, and the fatality rate of these conditions ranges from 60 to 90% [5,6,8,12]. Anisakiasis is the most common parasitic disease that causes eosinophilic granulomas in Japan, but it is distinctly different from strongyloidiasis. Anisakis infections often involve the stomach, rarely affect the colon, and generally cause acute abdomen. The associated granulomas are larger and sometimes form masses [23,24].

The findings of this study indicate that chronic strongyloidiasis can be diagnosed by identifying yellowish-white nodules in the cecum or right colon during colonoscopy and detecting filariform *Strongyloides* larvae within eosinophilic infiltrates in pathology specimens. We previously reported 10 cases of chronic strongyloidiasis in 2011 and described their colonoscopic and pathological features [5]. In addition, it was suggested that the re-invasion sites of the larvae may be primarily located in the cecum and proximal ascending colon. Although most reported cases of strongyloidiasis involved hyperinfection or disseminated infections, diagnosing chronic strongyloidiasis is crucial for preventing fatal disseminated strongyloidiasis [12].

The limitations of this study are as follows: 1. All cases were from Okinawa, Japan; 2. The number of cases was small. 3. No statistical analysis was performed. 4. No endoscopic or histological examination of the small intestine was performed.

## Conclusions

Colonoscopic findings in chronic strongyloidiasis showed multiple small yellowish-white nodules in the cecum and the proximal ascending colon. Histological findings revealed small, dense infiltrations of eosinophils in the lamina propria along with *Strongyloides* larvae. These findings are useful for diagnosing chronic strongyloidiasis and should lead to improved prognoses for patients.

In addition, it was suggested that the re-invasion site of larvae in chronic strongyloidiasis may be primarily located in the cecum and the proximal ascending colon.
